# Pharmacokinetics of Novel Plant Cell-Expressed Taliglucerase Alfa in Adult and Pediatric Patients with Gaucher Disease

**DOI:** 10.1371/journal.pone.0128986

**Published:** 2015-06-08

**Authors:** Richat Abbas, Glen Park, Bharat Damle, Raul Chertkoff, Sari Alon

**Affiliations:** 1 Pfizer, New York, NY, United States of America; 2 Target Health, New York, NY, United States of America; 3 Protalix Biotherapeutics, Carmiel, Israel; Baylor Research Institute, UNITED STATES

## Abstract

Taliglucerase alfa is a beta-glucocerebrosidase enzyme replacement therapy approved in the United States, Israel, and other countries for treatment of Type 1 Gaucher disease in adults, and is the first approved plant cell—expressed recombinant protein. In this report, taliglucerase alfa pharmacokinetics were assessed in adult and pediatric patients with Gaucher disease from separate multicenter trials of 30 Units/kg and 60 Units/kg doses infused every 2 weeks. Serial blood samples were obtained from adult patients following single-dose administration on day 1 (n = 26) and multiple doses at week 38 (n = 29), and from pediatric patients following administration of multiple doses of taliglucerase alfa for 10–27 months (n = 10). In both adult and pediatric patients, maximum plasma concentration (C_max_), area under the plasma concentration-time curve from time zero to last measureable concentration (AUC_0-t_), and from time zero to infinity (AUC_0-∞_) were higher after 60 Units/kg dose than 30 Units/kg dose. No tendency for accumulation or change in taliglucerase alfa pharmacokinetic parameters over time from day 1 to week 38 was observed with repeated doses of 30 or 60 Units/kg in adults. After multiple doses, mean (range) dose-normalized pharmacokinetic parameters were similar for adult versus pediatric patients receiving 60 Units/kg: C_max_ expressed in ng/mL/mg was 42.4 (14.5–95.4) in adults and 46.6 (34.4–68.4) in pediatric patients, AUC_0 t_ expressed in ng•h/mL/mg was 63.4 (26.3–156) in adults and 63.9 (39.8–85.1) in pediatric patients, t_1/2_ expressed in minutes was 34.8 (11.3–104) in adults and 31.5 (18.0–42.9) in pediatric patients and total body clearance expressed in L/h was 19.9 (6.25–37.9) in adults and 17.0 (11.7–24.9) in pediatric patients. These pharmacokinetic data extend the findings of taliglucerase alfa in adult and pediatric patients.

**Trial Registration:**

ClinicalTrials.gov. NCT00376168 (in adults); NCT01411228 (in children)

## Introduction

Gaucher disease (GD) is the most common lysosomal storage disorder with an estimated prevalence in the general population of ~1:50,000 [1]. A high degree of clinical heterogeneity is observed in patients, but all patients exhibit varying degrees of splenomegaly, hepatomegaly, thrombocytopenia, anemia, and skeletal pathology with the onset occurring during childhood through adulthood [2,3]. The disease is caused by mutations in the gene encoding beta-glucocerebrosidase, an enzyme that catalyzes the hydrolysis of glucosylceramide within lysosomes leading to accumulation of glucosylceramide primarily in macrophages and subsequent multi-system pathology. [2] Enzyme replacement therapy (ERT) is a treatment paradigm wherein deficient enzyme is replaced via infusion of active enzyme; it is the standard of care for patients with GD.[4] Successful treatment of GD requires targeting of the infused enzyme to lysosomes within macrophages. This occurs via uptake of appropriately glycosylated enzyme to permit efficient uptake by mannose receptors on the surface of macrophages [5–7].

A major advance in the care of patients with GD has been the development of processes to manufacture macrophage-targeted beta-glucocerebrosidase on a large scale [2]. Three ERTs are available for the treatment of Type 1 GD: imiglucerase, produced in a Chinese hamster ovary cell culture system [8,9]; velaglucerase alfa, produced in a human fibroblast cell system [10,11]; and taliglucerase alfa, an ERT produced in a plant cell–based expression system [12,13]. Taliglucerase alfa is the first US Food and Drug Administration–approved plant cell–expressed recombinant therapeutic protein [13,14]. It is indicated for treatment of adults with Type 1 GD in the United States, Israel, Australia, Canada, Chile, Brazil, and other countries, and is approved for treatment of pediatric patients in the United States, Australia, and Canada, and for hematologic manifestations in pediatric patients with Type 3 GD in Canada. The plant cell production system allows for generation of appropriately glycosylated glucocerebrosidase without the need for post-production enzymatic modification or mammalian-derived components in the production process [12].

Clinical trials have been conducted in adult and pediatric patients who were ERT-naïve and who had been switched from imiglucerase to taliglucerase alfa [15–17]. The approved dose of taliglucerase alfa for ERT-naïve adults is 60 Units/kg given every 2 weeks as a 60- to 120-minute intravenous infusion; dose adjustments may be made based on patient clinical achievements. The approved Units/kg dose for patients switching from imiglucerase is the same Units/kg dose of taliglucerase alfa [13].

To extend the findings of taliglucerase alfa, this report characterized the pharmacokinetics (PK) of 30 Units/kg and 60 Units/kg taliglucerase alfa in adult ERT-naïve patients with GD (single- and multiple-dose PK) and in pediatric patients with GD who were either ERT-naïve or had previously received imiglucerase treatment (multiple-dose PK).

## Methods

### Study design and patients

The objective of this analysis was to assess the PK of taliglucerase alfa in adult and pediatric patients from safety and efficacy studies PB-06-001 (adult patients) and PB-06-006 (pediatric patients) with GD using descriptive statistics.

The PK of taliglucerase alfa infused at nominal doses of 30 and 60 Units/kg body weight every 2 weeks in adults and pediatric patients with GD were evaluated using data from patients enrolled in 2 multicenter studies across 10 centers in Canada, Israel, Italy, Mexico, Spain, the United Kingdom, Chile, Paraguay, Serbia, and South Africa. Study protocols were reviewed and approved by the institutional review board/ethics committee at each study site. The studies were conducted in accordance with Good Clinical Practice guidelines. Investigators obtained informed, written consent from each adult patient and pediatric patient’s parent or guardian, and assent from each pediatric patient where applicable.

Adult PK were assessed in patients from a multicenter, 9-month, randomized pivotal trial (study PB-06-001; US National Institutes of Health www.clinicaltrials.gov Registration Identifier: NCT00376168) of taliglucerase alfa safety and efficacy in ERT-naïve, adult patients with GD [18]. This study was conducted from August 5, 2007, through September 11, 2009. Study design details have been published [15]. Briefly, patients were required to have a diagnosis of GD with leukocyte glucocerebrosidase activity ≤3 nmol/mg•hr, splenomegaly (8 times normal volume), and thrombocytopenia (<120,000/mm^3^) with or without anemia [15]. Patients who had received ERT or substrate inhibitor therapy within the previous 12 months were not allowed to enroll. Patients who had received ERT in the past could enroll provided that they had stopped receiving infusions for ≥12 months prior to study enrollment. Patients with severe neurological signs and symptoms characteristic of neuronopathic GD (complete ocular paralysis, overt myoclonus, or history of seizures) were excluded. Eligible adult patients were randomized to receive taliglucerase alfa 30 or 60 Units/kg (nominal dose) by intravenous infusion every 2 weeks. For consistency in the analysis, mean plasma concentrations of taliglucerase alfa were evaluated for patients receiving 120-minute infusions.

Pediatric PK were assessed in patients from an ongoing multicenter trial (study PB-06-006; NCT01411228) that enrolled pediatric participants (aged 2 to <18 years) with GD who had completed 12 months in protocol PB-06-005 (NCT01132690; ERT-naïve, randomized treatment with nominal doses of taliglucerase alfa 30 or 60 Units/kg for 12 months) or PB-06-002 (NCT00712348; open-label switch from imiglucerase to 9 months of treatment with taliglucerase alfa at the same dose as imiglucerase) [19–21]. Enrollment criteria for protocol PB-06-005 included a diagnosis of GD with leukocyte beta-glucocerebrosidase activity ≤30% of the mean activity of the reference range for healthy individuals, individual’s clinical need for ERT in the opinion of the investigator, no use of ERT in the past or no use of ERT during the previous 12 months and a negative anti-glucocerebrosidase antibody assay assessment, and no use of substrate inhibition therapy during the past 12 months [20]. Patients with neurological signs and symptoms characteristic of neuronopathic GD (other than long-standing oculomotor gaze palsy) were excluded [20].In study PB-06-005, the first patient was enrolled on October 11, 2010, and the last patient completed the study on April 10, 2012. Protocol PB-06-002 enrolled patients aged ≥2 years with a diagnosis of GD confirmed by enzymatic activity assay, and stable disease who had been receiving imiglucerase for ≥2 years and who had been on a stable maintenance regimen for the previous 6 months or more [21]. The first patient was enrolled on December 15, 2008, and the last patient completed the study on January 14, 2013. In protocol PB-06-006, these patients continued to receive taliglucerase alfa at the dose administered in the original study for an additional 24 months. Pediatric patients received taliglucerase alfa infusions over approximately 100 minutes every 2 weeks.

Blood samples for PK analysis in plasma were obtained at time 0 (before the start of the taliglucerase alfa infusion) and at 45, 70, 110, 125, 135, 150, 175, 200, and 225 minutes after the start of the infusion. In adult patients, samples were obtained following single-dose administrations on day 1 and multiple-dose administrations at week 38. In pediatric patients, samples were obtained after multiple-dose administration of taliglucerase alfa for at least 10 months.

### Bioanalytical methods

Blood samples were collected in tubes using K_3_EDTA as an anticoagulant. Plasma was separated by centrifugation at 1,500 x g for 15 minutes and stored at -70°C. Samples were sent to Midwest Bioresearch, LLC, a subsidiary of WIL Research Laboratories, LLC (Skokie, IL, USA), for PK sample analysis.

The concentrations of taliglucerase alfa in plasma were determined using a validated electrochemiluminescent (ECL) method with sulfo-tagged affinity purified anti-taliglucerase alfa. ECL was detected using an MSD Sector PR^TM^ 100 reader (Meso Scale Discovery, a division of Meso Scale Diagnostics, Rockville, MD) and the concentration of taliglucerase alfa was calculated using a 4-parameter curve fit equation. The lower limit of quantitation (LLOQ) was 7.8 ng/mL and the upper limit of quantitation was 1,000 ng/mL. The intra- and inter-assay precision was within the acceptance criterion of <30% coefficient of variation (range: 3.95% to 12.44%, 7.45% to 21.31%, respectively); the intra- and inter-assay accuracy was within the acceptance criterion of ±30% relative error (range: -19.94% to 20.05%, -12.48% to 29.16%, respectively).

### PK analyses and statistical calculations

PK parameters were determined using a noncompartmental analysis method. Concentration values reported below the LLOQ were assumed to be 0 ng/mL. Area under the plasma concentration versus time curve (AUC) from time 0 to the last measured concentration (AUC_0–t_) was calculated by linear trapezoidal estimation. Values for the elimination rate constant (k_e_) and the elimination half-life (t_1/2_) were considered reliable if the coefficient of determination (r^2^) was >0.8. AUC from time zero to infinity (AUC_0–∞_), total body clearance (CL), volume of distribution during the terminal elimination phase (V_z_), and volume of distribution at steady state (V_ss_) were considered reliable if the value for k_e_ was reliable and the percent extrapolation from AUC_0–t_ to AUC_0–∞_, was <25% for adult patients and <30% for pediatric patients. In study PB-06-001 (adult patients), the V_z_ was calculated, whereas in PB-06-006 (pediatric patients), the V_ss_ was calculated. For each nominal dose level (Units/kg) with 3 or more values for the parameter, descriptive statistics were calculated and reported for the maximum plasma concentration (C_max_), time to C_max_ (T_max_), AUC_0–t_, AUC_0–∞_, CL, V_ss_, and the normalized values. WinNonlin 5.0.1 and Phoenix WinNonlin 6.3 (Pharsight Corporation, a Certara company, St. Louis, MO, USA) were used for calculation of adult and pediatric PK parameters, respectively, with the exception of normalized parameter AUC_0-t_/dose, which was calculated using Microsoft Excel. Summary statistics by cohort (mean, median, standard deviation) were also calculated with Microsoft Excel.

## Results

### Demographics

Demographics for adult and pediatric patients in the PK analyses are listed in Table 1. The mean age of the adult patients was 37 years, the proportions of males and females were similar, and all but 1 patient was white. Twenty-six (30 Units/kg dose, n = 10; 60 Units/kg dose, n = 16) of the 31 adult patients in the intent-to-treat (ITT) population of study PB-06-001 were included in the day 1 PK analysis. Five patients (all were randomized to taliglucerase alfa 30 Units/kg) were excluded: 4 patients had infusions stopped and restarted on day 1, and 1 patient had day 1 samples that were incompletely collected and insufficient for analysis of PK parameters. Twenty-nine (30 Units/kg dose, n = 14; 60 Units/kg dose, n = 15) of the 31 adult patients in the ITT population completed study PB-06-001; all 29 patients were included in the week 38 PK analysis). The duration of multiple dosing with taliglucerase alfa in adults was 38 weeks.

**Table 1:** Baseline demographics of adult and pediatric patients.

		Adult Patients	Pediatric Patients
		(n = 29)	(n = 11)*
Characteristic	Category		
**Age, mean ± SD (range), years**		36.7 ± 12.2 (19–74)	12.27 ± 4.65 (4–18*)
**Sex, n (%)**	Female	14 (48)	4 (36)
	Male	15 (52)	7 (64)
**Weight, mean ± SD (range), kg**		68.6 ± 10.4 (50.0–93.0)	37.92 ± 17.87 (16.5–71.0)
**Duration of treatment, mean ± SD (range)**		38 weeks for all patients	21.36 ± 5.94 months (10–27 months)

SD, standard deviation.

*One pediatric patient from study PB-06-002 was 18 years of age prior to PK sample collection and was excluded from the PK analysis. ^†^Two pediatric patients from study PB-06-002 did not provide religion information.

Fifteen pediatric patients were enrolled in study PB-06-006. The mean age was 12 years, approximately two thirds were male, and all were white. Ten of these patients were included in the pediatric PK analysis. Three patients were excluded because the protocol amendment for the PK analysis was not approved in the patients’ country, 1 patient was excluded from the PK analysis because of parental refusal to grant informed consent, and 1 patient was excluded because she reached the age of 18 years before sampling was scheduled to take place. Six of the 10 pediatric patients in this PK analysis received approximately 30 Units/kg (range, 20 to 35 Units/kg) and 4 received approximately 60 Units/kg (range, 49 to 60 Units/kg). The duration of multiple dosing with taliglucerase alfa in individual pediatric patients ranged from 10 to 27 months.

### PK analysis in adult patients

**Fig 1:**
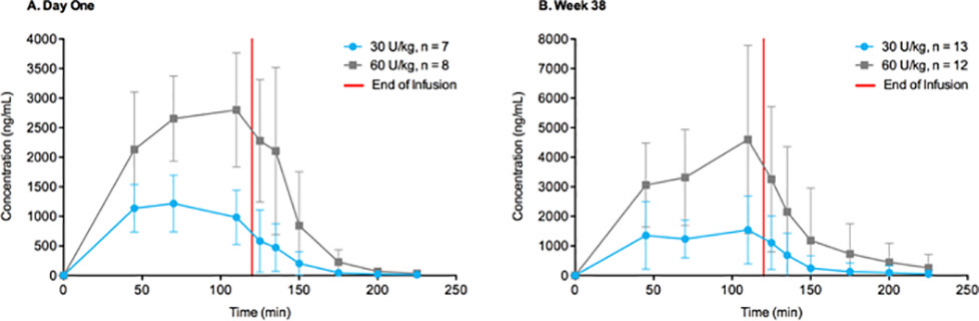
Taliglucerase alfa plasma concentration in adult patients. Mean plasma concentration-versus-time curve of taliglucerase alfa in adult patients for 120-minute infusions showing dose-dependent increase (linear plot): a) on day 1; b) at week 38. Abbreviation: U/kg, Units/kg. Error bars represent standard deviations.

The time courses for taliglucerase alfa plasma concentration are shown in Fig 1 for day 1 (single-dose treatment) and week 38 (multiple-dose treatment). PK parameters for adult patients are summarized in Table 2. Measures of exposure (C_max_, AUC_0–t_, and AUC_0–∞_) were higher after the 60 Units/kg dose than the 30 Units/kg dose on day 1 and at week 38, although there was an overlap in the ranges of the values. The mean T_max_ ranged from 82.5–95.0 min, mean t_1/2_ ranged from 25.0–34.8 min, mean clearance rates (CL) ranged from 19.9–30.7 L/h, and mean V_z_ ranged from 11.7–17.5 L on day 1 and week 38. There was no apparent dependence of mean T_max_, t_1/2_, CL, or V_z_ on dose or sampling day. No tendency for accumulation or change in taliglucerase alfa PK over time from day 1 to week 38 was observed with repeated doses of 30 or 60 Units/kg.

**Table 2:** Summary of PK parameters of taliglucerase alfa in adult patients.

PK Parameter	Taliglucerase Alfa 30 U/kg		Taliglucerase Alfa 60 U/kg	
	Day 1 (n = 10)	Week 38 (n = 14)	Day 1 (n = 16)	Week 38 (n = 15)
**C_max_, ng/mL, mean ± SD**	1,556 ± 742	1,656 ± 1,116	4,250 ± 2,230	5,153 ± 3,099
**Median**	1,504	1,382	3,650	4,565
**Range**	637–3,275	720–4,989	1,792–10,351	1,834–12,504
**T_max_, min, mean ± SD**	82.5 ± 42.1	85.0 ± 33.1	86.6 ± 28.4	95.0 ± 28.8
**Median**	70.0	110	75.0	110
**Range**	45–175	45–125	45–135	45–135
**AUC_0–t_, ng**•**hr/mL, mean ± SD**	2,229 ± 669	2,654 ± 2,130	6,349 ± 2,200	7,665 ± 4,578
**Median**	2,441	1,989	6,350	6,751
**Range**	807–3,082	1,002–9,546	2,877–10,077	2,545–20,496
**AUC_0–∞_, ng**•**hr/mL, mean ± SD**	2,244 ± 674	2,706 ± 2,270	6,383 ± 2,229	7,814 ± 5,157*
**Median**	2,459	2,007	6,372	6,459
**Range**	810–3,119	1,007–10,092	2,885–10,265	2,548–21,020
**t_1/2_, min, mean ± SD**	25.9 ± 11.8	25.1 ± 15.5	25.0 ± 10.1	34.8 ± 22.9*
**Median**	23.6	18.9	21.9	28.7
**Range**	9.95–42.4	9.20–57.9	13.3–43.7	11.3–104
**CL,L/hr, mean ± SD**	29.4 ± 13.9	30.7 ± 14.5	20.5 ± 7.1	19.9 ± 9.6*
**Median**	23.2	30.5	19.7	18.5
**Range**	16.8–56.4	6.79–68.0	10.0–35.6	6.25–37.9
**V_z_,L, mean ± SD**	17.5 ± 11.1	16.8 ± 12.7	11.7 ± 4.5	14.4 ± 6.8*
**Median**	13.8	12.7	12.5	13.8
**Range**	6.19–45.9	6.95–55.3	5.69–20.4	3.91–24.8

AUC_0–t_, area under the plasma concentration-time curve from time zero to the last sampling time; AUC_0–∞_, area under the plasma concentration-time curve extrapolated from time zero to infinity; CL, total body clearance; C_max_, maximum plasma concentration; PK, pharmacokinetics; SD, standard deviation; T_max_, time of maximum plasma concentration; t_1/2_, elimination half-life; U/kg, Units/kg; V_z_, volume of distribution during terminal elimination phase.

*n = 14.

### PK analysis in pediatric patients

**Fig 2:**
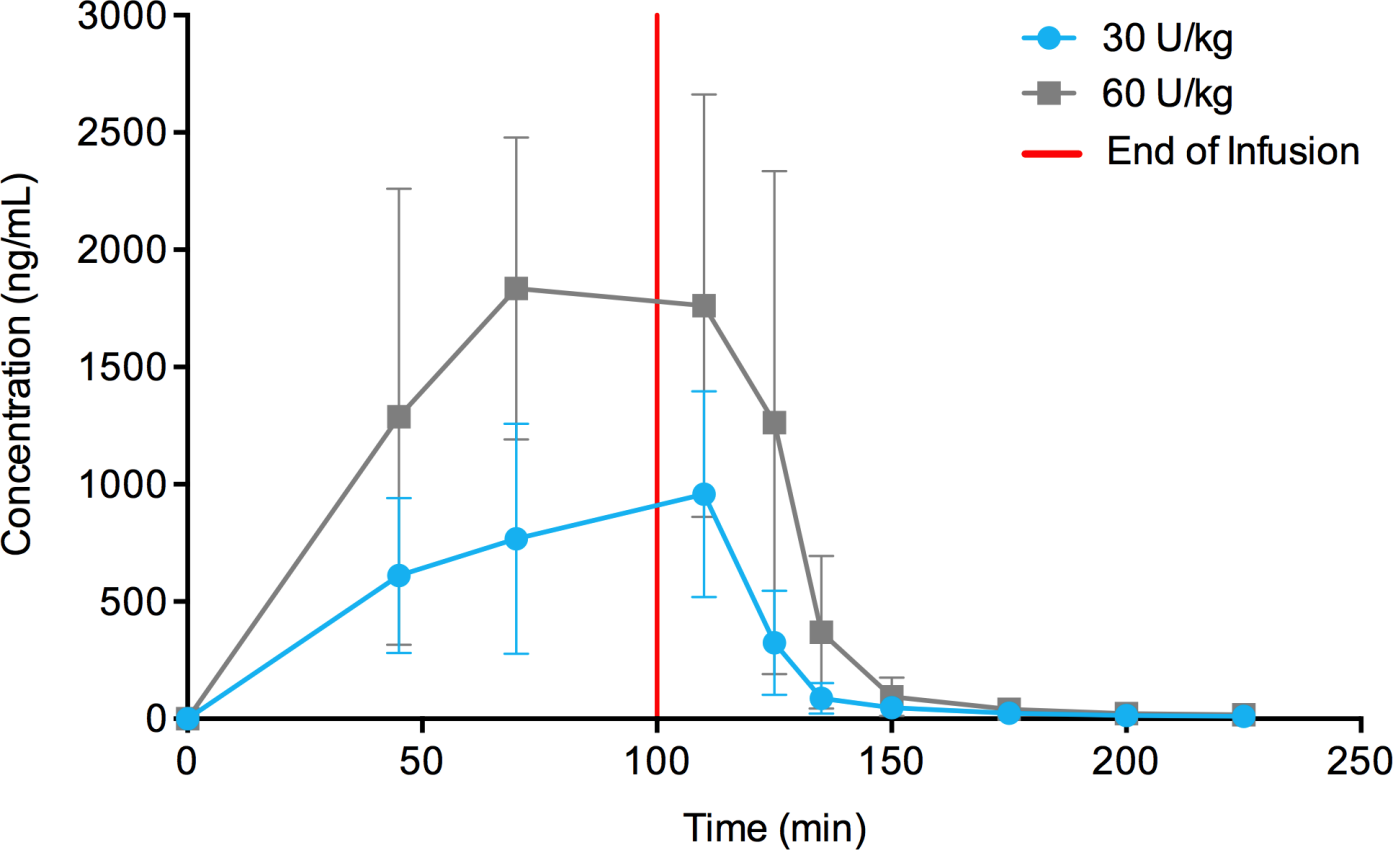
Taliglucerase alfa plasma concentration in pediatric patients. Mean plasma concentration-versus-time curve of taliglucerase alfa in pediatric patients for approximately 100-minute infusions showing dose-dependent increase (linear plot). Abbreviation: U/kg, Units/kg. Error bars represent standard deviations.

The time course of taliglucerase alfa plasma concentration is shown in Fig 2 for pediatric patients following at least 10 months of multiple-dose treatment. PK parameters for pediatric patients are summarized in Table 3. C_max_, AUC_0–t_, and AUC_0–∞_ were higher at the 60 Units/kg than 30 Units/kg dose. Mean values for T_max_ and t_1/2_ were similar for the taliglucerase alfa at 30 Units/kg and 60 Units/kg doses.

**Table 3:** Summary of PK parameters of taliglucerase alfa in pediatric patients.

PK Parameter	Taliglucerase Alfa 30 U/kg (n = 6)	Taliglucerase Alfa 60 U/kg (n = 4)
**Actual dose, U/kg, mean ± SD**	28.2 ± 5.7	54.5 ± 4.6
**Median**	27.4	54.9
**Range**	19.9–35.3	48.5–59.6
**C_max_, ng/mL, mean ± SD**	1,084 ± 409	2,044 ± 605
**Median**	1,121	1,953
**Range**	389–1,539	1,518–2,754
**T_max_, min, mean ± SD**	90.0 ± 21.9	83.8 ± 32.0
**Median**	90.0	90.0
**Range**	70.0–110	45.0–110
**AUC_0–t_, ng**•**hr/mL, mean ± SD**	1,336 ± 527	2,947 ± 1,371
**Median**	1,491	2,969
**Range**	527–1,932	1,593–4,256
**AUC_0–∞_, ng**•**hr/mL, mean ± SD**	1,349 ± 531	2,962 ± 1,378
**Median**	1,496	2,984
**Range**	535–1,969	1,606–4,273
**t_1/2_, min, mean ± SD**	34.8 ± 17.4	31.5 ± 11.6
**Median**	31.9	32.5
**Range**	12.9–56.8	18.0–42.9
**CL, L/hr, mean ± SD**	25.5 ± 10.0	17.0 ± 6.1
**Median**	27.4	15.8
**Range**	10.9–37.8	11.7–24.9
**V_ss_, L, mean ± SD**	16.4 ± 11.1	10.7 ± 7.8
**Median**	13.7	8.8
**Range**	3.75–35.6	3.75–21.4

AUC_0–t_, area under the plasma concentration-time curve from time zero to the last sampling time; AUC_0–∞_, area under the plasma concentration-time curve extrapolated from time zero to infinity; CL, total body clearance; C_max_, maximum plasma concentration; PK, pharmacokinetics; SD, standard deviation; T_max_, time of maximum plasma concentration; t_1/2_, elimination half-life; U/kg, Units/kg; V_ss_, volume of distribution during steady-state.

### Comparison of PK parameters in adult and pediatric patients

Following repeated infusions of taliglucerase alfa (week 38 for adult patients and at least 10 months for pediatric patients), mean t_1/2_ and CL values in pediatric patients (Table 4) were similar to those observed in adult patients. Dose-normalized (mg) exposure was also comparable in pediatric and adult patients (Table 4).

**Table 4:** Comparative summary of PK parameters for taliglucerase alfa following multiple-dose intravenous infusion of 30 or 60 U/kg in adult and pediatric patients with Gaucher disease.

Parameter	Adult Patients at Week 38		Pediatric Patients at ≥10 Months	
	30 U/kg (n = 14)	60 U/kg (n = 15)	30 U/kg (n = 6)	60 U/kg (n = 4)
NC_max_ (ng/mL)/mg, mean	26.8	42.4*	37.0	46.6
Range	10.5-72.8	14.5-95.4	22.4-63.6	34.4-68.4
NAUC_0-t_ (ng•h/mL)/mg, mean	42.2	63.4	46.4	63.9
Range	14.6-139	26.3-156	26.0-91.7	39.8-85.1
t_1/2_ (min), mean	25.1	34.8	34.8	31.5
Range	9.2-57.9	11.3-104	12.9-56.8	18.0-42.9
CL (L/h), mean	30.7	19.9	25.5	17.0
Range	6.79-68.0	6.25-37.9	10.9-37.8	11.7-24.9

CL, total body clearance; NAUC_0–t_, area under the plasma concentration-time curve from time zero to the last sampling time normalized by dose; NC_max_, maximum plasma concentration normalized by dose; PK, pharmacokinetics; SD, standard deviation; t_1/2_, elimination half-life; U/kg, Units/kg.

## Discussion

The single- and multiple-dose PK data of taliglucerase alfa in adult patients with GD and multiple-dose PK data in pediatric patients with GD have been presented in this report. For both patient populations, exposure to taliglucerase alfa, as measured by C_max_, AUC_0–t_, and AUC_0–∞_, was higher after the 60 Units/kg dose than the 30 Units/kg dose. In addition, mean values for T_max_ and t_1/2_ were similar for taliglucerase alfa 30 and 60 Units/kg. No tendency for accumulation or change in taliglucerase alfa PK over time from day 1 to week 38 was observed with repeated doses of 30 or 60 Units/kg in adults. In pediatric patients, dose-normalized exposure indicated dose proportionality between 30 and 60 Units/kg.

Previously, Aviezer et al [22] reported an earlier, Phase 1 study of the PK of taliglucerase alfa in healthy adult volunteers (n = 6) who received escalating doses administered intravenously at separate clinic visits (15 Units/kg on day 8, 30 Units/kg on day 15, and 60 Units/kg on day 22). Maximum plasma concentration of taliglucerase alfa was reached by approximately 80 min after the start of infusion. Measures of exposure to taliglucerase alfa showed dose dependence. The range of values for t_1/2_ (8–32 min) and CL (0.8–3.4 mL/min/kg) were comparable to those for adult patients in this analysis.

There is continuing research and clinical interest in ERT PK and pharmacodynamics (PD) due to unanswered questions related to dosing, long-term treatment, tissue targeting, and in vivo half-life in specific tissues [23,24]. For example, Xu et al [23] described the comparative PK and PD of imiglucerase and velaglucerase alfa in liver, spleen, and lung using standard biochemical methodology in a mouse model of GD. Phenix et al [24], taking a different approach, reported the development of the method for synthesizing a radiolabeled beta-glucocerebrosidase substrate analog that was suitable for positron emission tomography imaging and analysis of the biodistribution of beta-glucocerebrosidase–based ERT in a mouse model.

The present analysis was limited by small numbers of patients, the use of multiple studies with non-identical designs, inclusion of patients with different therapeutic histories (e.g., patients switched from imiglucerase to taliglucerase alfa and patients continuing on taliglucerase alfa), and the absence of single-dose data for pediatric patients.

In summary, this report provides an extensive set of data and characterization of the pharmacokinetics of taliglucerase alfa, the first plant cell–expressed ERT for GD, across pediatric and adult patient populations.

## Supporting Information

S1 File Supplementary Methods. (DOCX)

## References

[1] Meikle PJ, Hopwood JJ, Clague AE, Carey WF (1999) Prevalence of lysosomal storage disorders. JAMA 281: 249–54. 9918480 10.1001/jama.281.3.249

[2] Grabowski GA, Petsko GA, Kolodny EH. Gaucher disease In: Scriver CR, Beudet AL, Sly WS, eds. The Metabolic and Molecular Basis of Inherited Disease. New York, NY: McGraw-Hill, 2010:1–143.

[3] Grabowski GA, Kolodny EH, Weinreb NJ, Rosenbloom BE, Prakash-Cheng A, Kaplan P, et al Gaucher Disease: Phenotypic and Genetic Variation In: Valle D, Beaudet AL, Vogelstein B, et al., eds. The Online Metabolic & Molecular Bases of Inherited Disease. New York, NY: McGraw Hill Global Education, 2010.

[4] Cox TM (2013) Competing for the treasure in exceptions. Am J Hematol 88: 163–5. 10.1002/ajh.23399 23400870

[5] Stahl PD, Rodman JS, Miller MJ, Schlesinger PH (1978) Evidence for receptor-mediated binding of glycoproteins, glycoconjugates, and lysosomal glycosidases by alveolar macrophages. Proc Natl Acad Sci U S A 75: 1399–403. 274729 10.1073/pnas.75.3.1399 PMC411479

[6] Barton NW, Brady RO, Dambrosia JM, Di Bisceglie AM, Doppelt SH, Hill SC, et al (1991) Replacement therapy for inherited enzyme deficiency—macrophage-targeted glucocerebrosidase for Gaucher's disease. N Engl J Med 324: 1464–70. 2023606 10.1056/NEJM199105233242104

[7] Friedman B, Vaddi K, Preston C, Mahon E, Cataldo JR, McPherson JM (1999) A comparison of the pharmacological properties of carbohydrate remodeled recombinant and placental-derived beta-glucocerebrosidase: implications for clinical efficacy in treatment of Gaucher disease. Blood 93: 2807–16. 10216074

[8] Grabowski GA, Barton NW, Pastores G, Dambrosia JM, Banerjee TK, McKee MA, et al (1995) Enzyme therapy in type 1 Gaucher disease: comparative efficacy of mannose-terminated glucocerebrosidase from natural and recombinant sources. Ann Intern Med 122: 33–9. 7985893 10.7326/0003-4819-122-1-199501010-00005

[9] Cerezyme [package insert]. (2009) Cambridge, MA: Genzyme Corporation.

[10] Zimran A, Loveday K, Fratazzi C, Elstein D (2007) A pharmacokinetic analysis of a novel enzyme replacement therapy with Gene-Activated human glucocerebrosidase (GA-GCB) in patients with type 1 Gaucher disease. Blood Cells Mol Dis 39: 115–8. 17391996 10.1016/j.bcmd.2007.02.008

[11] VPRIV [package insert]. (2013) Lexington, MA: Shire Human Genetic Therapies, Inc.

[12] Shaaltiel Y, Bartfeld D, Hashmueli S, Baum G, Brill-Almon E, Galili G, et al (2007) Production of glucocerebrosidase with terminal mannose glycans for enzyme replacement therapy of Gaucher's disease using a plant cell system. Plant Biotechnol J 5: 579–90. 17524049 10.1111/j.1467-7652.2007.00263.x

[13] Elelyso [package insert]. (2014) New York, NY: Pfizer Labs.

[14] Fox JL (2012) First plant-made biologic approved. Nat Biotechnol 30: 472.

[15] Zimran A, Brill-Almon E, Chertkoff R, Petakov M, Blanco-Favela F, Terreros Munoz E, et al (2011) Pivotal trial with plant cell-expressed recombinant glucocerebrosidase, taliglucerase alfa, a novel enzyme replacement therapy for Gaucher disease. Blood 118: 5767–73. 10.1182/blood-2011-07-366955 21900191

[16] Zimran A, Gonzalez-Rodriguez DE, Abrahamov A, Elstein D, Heitner R, Paz A, et al (2012) A multicenter, double-blind, randomized safety and efficacy study of two dose levels of taliglucerase alfa in pediatric patients with Gaucher disease [abstract 2140]. Blood 120: 1–2. 10.1182/blood-2012-04-425306 22767572

[17] Pastores GM, Petakov M, Giraldo P, Rosenbaum H, Szer J, Deegan PB, et al. (17 June 2014) A phase 3, multicenter, open-label, switchover trial to assess the safety and efficacy of taliglucerase alfa, a plant cell expressed reconbinant human glucocerebrosidase, in adult and pediatric patients with Gaucher disease previously treated with imiglucerase. Blood Cells Mol Dis [Epub ahead of print]. 10.1016/j.bcmd.2014.05.004 24950666

[18] A phase III trial to assess the safety and efficacy of plant cell expressed GCD in patients with Gaucher disease NCT00376168. (Last update: 25 July 2012.) National Institutes of Health Available: http://www.clinicaltrials.gov/ct2/show/NCT00376168. Accessed 29 May 2014. 10.1016/j.bcmd.2014.05.004

[19] A multicenter extension study of taliglucerase alfa in pediatric subjects with Gaucher disease. NCT01411228. (Last update: 13 August 2013.) National Institutes of Health Available: http://clinicaltrials.gov/ct2/show/NCT01411228?term=PB-06-005&rank=1. Accessed 27 January 2014.

[20] A safety and efficacy study of two dose levels of taliglucerase alfa in pediatric subjects with Gaucher disease [NCT01132690]. (Last update: 10 June 2013.) National Institutes of Health Available: http://clinicaltrials.gov/ct2/show/NCT01132690?term=NCT01132690&rank=1. Accessed 27 January 2014. 10.1016/j.bcmd.2014.10.002

[21] Switchover trial from imiglucerase to plant cell expressed recombinant human glucocerebrosidase [NCT00712348]. (Last update: 10 June 2013.) National Institutes of Health Available: http://clinicaltrials.gov/ct2/show/NCT00712348?term=nct00712348&rank=1. Accessed 27 January 2014. 10.1016/j.bcmd.2014.05.004

[22] Aviezer D, Brill-Almon E, Shaaltiel Y, Hashmueli S, Bartfeld D, Mizrachi S, et al (2009) A plant-derived recombinant human glucocerebrosidase enzyme—a preclinical and phase I investigation. PLoS One 4: e4792 10.1371/journal.pone.0004792 19277123 PMC2652073

[23] Xu YH, Sun Y, Barnes S, Grabowski GA (2010) Comparative therapeutic effects of velaglucerase alfa and imiglucerase in a Gaucher disease mouse model. PLoS One 5: e10750 10.1371/journal.pone.0010750 20505772 PMC2873993

[24] Phenix CP, Rempel BP, Colobong K, Doudet DJ, Adam MJ, Clarke LA, et al (2010) Imaging of enzyme replacement therapy using PET. Proc Natl Acad Sci U S A 107: 10842–7. 10.1073/pnas.1003247107 20534487 PMC2890769

