# Supplementary material for: Pharmacokinetics of Novel Plant Cell-Expressed Taliglucerase Alfa in Adult and Pediatric Patients with Gaucher Disease
Source: PLoS One. 2015 Jun 8;10(6):e0128986. doi: 10.1371/journal.pone.0128986 (PMC4459956; doi:10.1371/journal.pone.0128986)
Supplement: S1 File — (DOCX) [file pone.0128986.s001.docx]

**SUPPLEMENTARY METHODS**

Study protocols were reviewed and approved by the following institutional review board/ethics committee at each study site: Canada—Mount Sinai Hospital Research Ethics Board, Toronto, Ontario, Canada; Israel—Helsinki Committee, Hebrew University School of Medicine, Shaare Zedek Medical Center, Jerusalem, Israel; Helsinki Committee, Rambam Medical Center, Haifa, Israel; Italy—Comitato Etico Azienda Policlinico Umberto I, Rome, Italy; Mexico—Comision Nacional de Investigación Científica Instituto Mexican del Seguro Social, Mexico D.F. C.P.; Spain—Comité Etico de Investigacion Clinica de Aragon, Zaragoza, Spain; United Kingdom—Moorfields and Whittington Research Ethics Office, London, UK; Chile—Comité de Ética, Facultad de Medicina, Pontificia Universidad Católica de Chile, Santiago, Chile; Comité Etico Cientifico, Gobierno de Chile, Ministerio de Salud, Santiago, Chile; Paraguay—Comite de Etica de Investigacion, Facultad de Ciencias Medicas, Universidad Nacional de Asuncion, Asuncion, Paraguay; Serbia—Ethics Committee of Clinical Center of Serbia, Belgrade, Serbia; South Africa—Pharma-Ethics Independent Research Ethics Committee, Lyttelton Manor, South Africa.
